# Can cutaneous telangiectasiae as late normal-tissue injury predict cardiovascular disease in women receiving radiotherapy for breast cancer?

**DOI:** 10.1038/sj.bjc.6605182

**Published:** 2009-07-14

**Authors:** G A Tanteles, J Whitworth, J Mills, I Peat, A Osman, G P McCann, S Chan, J G Barwell, C J Talbot, R P Symonds

**Affiliations:** 1 Department of Genetics, University of Leicester, Leicester LE1 5WW, UK; 2 Department of Cancer Studies and Molecular Medicine, University Hospitals of Leicester, Level 2, Osborne Building, Leicester Royal Infirmary, Leicester LE1 5WW, UK; 3 Department of Cardiology, University Hospitals of Leicester, Glenfield Hospital, Groby Road, Leicester LE1 5WW, UK; 4 Department of Clinical Oncology, Nottingham University Hospitals NHS Trust (City Hospital Campus), Nottingham, UK

**Keywords:** breast cancer, late-radiation damage, CVD, telangiectasiae

## Abstract

**Background:**

Overall, ∼5% of patients show late normal-tissue damage after radiotherapy with a smaller number having a risk of radiation-induced heart disease. Although the data are conflicting, large studies have shown increased risks of cardiovascular disease (CVD) for irradiated patients compared with non-irradiated ones, or for those treated to the left breast or chest wall compared with those treated to the right. Cutaneous telangiectasiae as late normal-tissue injury have so far only been regarded as a cosmetic burden.

**Methods:**

The relationship between late normal-tissue radiation injury phenotypes in 149 irradiated breast cancer patients and the presence of cardiovascular disease were examined.

**Results:**

A statistically significant association between the presence of skin telangiectasiae and the long-term risk of CVD was shown in these patients (*P*=0.017; Fisher's exact test).

**Interpretation:**

This association may represent initial evidence that telangiectasiae can be used as a marker of future radiation-induced cardiac complications. It could also suggest a common biological pathway for the development of both telangiectasiae and CVD on the basis of a genetically predisposed endothelium. To our knowledge this is the first reported study looking at this association.

Virtually all breast cancer patients treated with breast conservation and patients at high risk of recurrence after mastectomy receive radiotherapy. The benefits of radiation therapy are not however complication free. There is a wide spectrum of normal-tissue reactions, and as life expectancy of cancer patients improves, these are increasingly of clinical importance. Tissue toxicity may range from asymptomatic changes in tissue structure and function, to severe cosmetic disfigurement and life-altering changes in organ function (Bentzen *et al*, 2003). The effects of radiotherapy can be divided into early/acute or late, depending on whether they occur within or after 90 days after radiation treatment (van der Kogel, 1993). Fibrosis, telangiectasiae and atrophy are examples of common late normal-tissue manifestations (O’Sullivan and Levin, 2003).

Radiotherapy substantially reduces the risk of local recurrence after surgery, with a modest reduction in cancer mortality offset by an increase in contralateral breast cancer and cardiac disease, which is more marked in older trials. Many of the techniques used involved some unavoidable irradiation of the heart leading to a 27% (95% confidence interval (CI) 13–41%) increase in mortality from heart disease and reducing the beneficial effect of radiotherapy on overall survival (Clarke *et al*, 2005). The cardiovascular mortality risk in patients receiving radiotherapy for left-sided breast cancer tends to become apparent after more than 10 years of follow-up (Darby *et al*, 2003). It seems that this risk is side and technique specific and is particularly associated with irradiation of the internal mammary nodes (HR=1.9, 95% CI 1.0–3.3) (Paszat *et al*, 2007). Evidence also exists of a significant increase in the relative risk for myocardial infarction (MI) for women who received adjuvant radiotherapy for left-sided compared with right-sided lesions (Rutqvist and Johansson, 1990; Paszat *et al*, 1998). All cardiac structures including the pericardium, myocardium, valves, conduction system and coronary arteries are amenable to radiation damage, and a common pathophysiological pathway appears to be that of microcirculatory damage (with the exception of valvular disease as valves are avascular) (Stewart *et al*, 1995).

More recently, a greater number of patients received treatment to the intact breast without parasternal internal mammary node irradiation, which reduces the cardiac irradiated volume. Although the cardiac dose from left-tangential radiotherapy has also decreased considerably over the past few decades, part of the heart still receives >20 Gy for approximately half of those with left-sided tumours. The cardiac dose for right-sided patients is generally from scattered irradiation alone (Taylor *et al*, 2008).

A wide range of additional risk factors have been associated with an increased risk for cardiovascular morbidity and mortality after breast cancer radiotherapy. These include irradiated heart volume (Gyenes *et al*, 1997), total radiation dose (Gyenes *et al*, 1998) and fractionation (Paszat *et al*, 1999), although the latter has not been consistently shown (Marhin *et al*, 2007; Nilsson *et al*, 2009). There seems to be an interaction between radiation-related cardiac damage and cardiotoxic drugs such as anthracyclines (Bonneterre *et al*, 2004), whereas acute cardiotoxicity following combination of left-sided internal mammary chain irradiation and concurrent trastuzumab use has not been clearly shown (Belkacemi *et al*, 2008; Shaffer *et al*, 2009). A low overall incidence of cardiovascular adverse effects has also been shown with the use of aromatase inhibitors and tamoxifen (Mouridsen *et al*, 2007). In terms of coexisting risk factors for developing radiation-induced heart disease (RIHD), there is a significant interaction between pre-existing hypertension, left-breast irradiation and the development of coronary artery disease (Harris *et al*, 2006).

In an attempt to associate genotypes with phenotypes, our group examined the relationship between late normal-tissue radiation injury in breast cancer patients, early acute radiation reactions and genotype (Giotopoulos *et al*, 2007). Patients were genotyped at functional single nucleotide polymorphisms (SNPs) in various candidate genes. As the power of genetic studies investigating the genotype–phenotype correlations is absolutely dependent on the precise definition of the phenotype, the Late Effects of Normal Tissue-Subjective Objective Management Analytical (LENT-SOMA) scale was used to evaluate radiation injury (Pavy *et al*, 1995). This scale provides both subjective and objective analyses and a detailed and specific description of the nature (phenotype) and severity of the injury(s) that are individually scored. Results showed that homozygosity (TT) for the *TGFb1* (C-509T) gene promoter polymorphism confers a 15-fold increased risk of fibrosis after radiotherapy (*P*=3x10^−6^) compared with (CC) homozygotes, thus confirming previous independent analyses (Quarmby *et al*, 2003; Andreassen *et al*, 2005). In addition, a 15 Gy electron boost and/or the inheritance of X-ray repair cross-complementing 1 (*XRCC1*) (R399Q) SNP contributed to the risk of telangiectasiae. These data suggested distinct underlying genetic and radiobiological pathways responsible for these side effects.

On the basis of the data from our previous study cohort, we hypothesised a potential association between the presence of cutaneous telangiectasiae as a late normal-tissue injury after radiotherapy for breast cancer and an increase in the risk for cardiovascular disease.

## Materials and methods

### Patients and methods

This report follows a previous study published by this group (Giotopoulos *et al*, 2007). The study had been undertaken with the participation of patients attending the breast cancer clinics in Leicester Royal Infirmary and Glenfield Hospital, Leicester, UK. The Oncology Department provides services for a population of just below a million observing over 3500 new cases per year of which 750 are newly diagnosed patients with breast cancer. All patients with breast cancer having completed adjuvant treatment are followed up annually for a minimum of 5 years.

**Figure 1:**
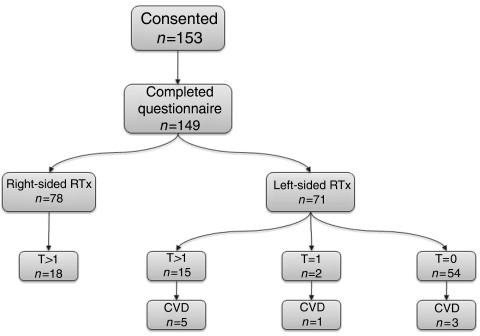
Flowchart showing patients included in the analysis. CVD, cardiovascular disease; RTx, radiation therapy; T, telangiectasia score.

Tumour-free breast cancer patients were sequentially recruited by these breast cancer clinics. A total of 153 patients gave written informed consent before entry into the study, and underwent an examination of the affected area, and the recognised features of late radiation effects were scored by the SOMA scale using sight and/or palpation. Of these, fully completed questionnaires were obtained for the majority of patients (*n*=149) (Figure 1). All were treated more than 4 years previously (median follow-up from time of treatment for the entire group was 6.1 years). Radiotherapy (6 MV photons) was given either after mastectomy or wide local excision (WLE) using tangential opposed fields. Initial surgery was either a WLE with nodal sampling/clearance (*n*=101) or mastectomy (*n*=45) plus axillary node dissection. Three patients had inoperable tumours.

A variety of radiotherapy dose-fractionation schedules were used (see Table 1). Two patients discontinued treatment early because of unexpected severe acute reactions. All patients who received an electron boost had a dose of 15 Gy in five fractions in 1 week using 8–12 MeV electrons to the tumour bed. The irradiated area on these patients was similar (between 36 and 80 cm^2^). Patients were asked about potential radiosensitising comorbid diseases such as diabetes or collagen vascular disorders. They also completed a questionnaire regarding cardiac symptoms and diagnoses.

Cardiovascular disease (CVD) was defined by symptoms and corresponding investigations that showed an abnormality in cardiac function or structure. Vascular disease such as hypertension was not part of the CVD definition, although it was considered as a risk factor for it. In this definition we included coronary artery disease, atrial or ventricular dilatation, arrhythmias and congestive cardiac failure. We excluded congenital heart disease or acquired/secondary structural disease. Patients with known congenital (cardiac shunts, previous corrective surgery or persisting structural abnormalities such as valvular heart disease) or acquired/secondary structural heart disease (rheumatic valve disease, mixed aortic valve disease, Coronary Artery Bypass Graft (CABG)) before radiotherapy were excluded from the current analysis following a detailed review of all available medical information by a Consultant Cardiologist (GPM). Cardiovascular symptoms/diagnoses were checked for all the recruited patients using a variety of information systems within the University Hospitals Leicester, which provide all secondary care assessment of patients with known or suspected cardiac disease within Leicestershire. Patients who had been investigated with an echocardiogram were highlighted within the Cardiac Investigations’ own record system and the report obtained. The online radiology reports system was screened to highlight any other relevant imaging such as cardiac perfusion scans or coronary angiography. In addition, all patient records on the Hospital Information Support System (HISS) medical records/clinical coding system were examined to check for clinical codes relating to cardiac diagnoses. All the medical symptoms reported in oncology follow-up appointments were reviewed using the electronic patient record system (MAISY – Compucorp, Watford, Hertfordshire, UK). Death certificates of deceased patients were obtained to check for causes of death related to cardiac damage. Finally, any patient with a positive finding in any of these modalities was investigated further with a full review of their written medical records.

**Figure 2:**
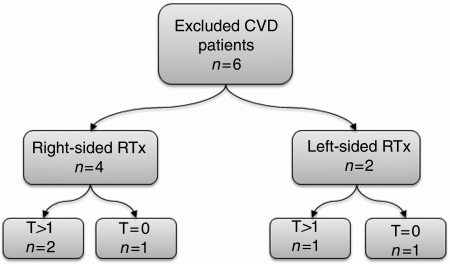
Flowchart showing excluded patients with documented cardiovascular disease. One patient with a telangiectasia score of 1 is not shown. CVD, cardiovascular disease; RTx, radiation therapy.

A total of fifteen patients with cardiac disease requiring referral or investigations in secondary or tertiary care and who fulfilled the inclusion criteria were identified. Six patients were excluded on the basis of having pre-existing cardiac disease (Figure 2). Two of these patients received left- and four received right-sided radiotherapy. Reasons for exclusion included: cardiomegaly and pulmonary congestion before radiotherapy treatment (one patient), CABG pre-radiotherapy treatment (one patient), rheumatic valve disease (one patient) and congenital heart disease (three patients).

To assess whether the CVD patients (*n*=9) had excessive heart tissue irradiated, simulator planning films from all the patients treated with left-sided radiotherapy were reviewed by three investigators (RPS, JM, GAT). Accurately interpretable information from planning films could only be obtained for 60 of the 71 left-sided patients. Of these, 42 patients had some cardiac outline within the tangential field (defined as ‘in-field’) and 18 did not. The heart in right-sided patients was automatically regarded as ‘out-of-treatment field’ (*n*=78), thus giving an ‘out-of-field’ denominator of 96 and a total for the group of 138. The maximum heart distance (MHD) was measured as a proxy for irradiated volume with established level of agreement. The MHD was measured between the posterior (mediolateral) field border to the most distant heart contour in the beam's-eye view of a tangential treatment beam.

On the basis of the available information, the denominator slightly differed between the analysis groups. Treatment side information was available for 149 patients. The presence or absence of telangiectasiae was recorded for 149 patients; however, as 12 patients with a telangiectasia SOMA score of 1 were excluded in an attempt to reduce inter-examiner bias, the analysis denominator became 137 (see Results section).

### Statistical analysis

The Fisher's exact test was used to examine the relationship between the various variables as well as the relationship between phenotype and genotype. To test whether hypertension was an independent risk factor, binary logistic regression was used. The Statistical Package for the Social Sciences (SPSS 16.0, Chicago, Illinois, USA) software package was used for the analysis.

### Genotyping

A full description of the genotyping methods is included in our previous paper (Giotopoulos *et al*, 2007).

## Results

Of the patients with available data (*n*=149), 6% (9 of 149) were identified as having cardiovascular disease between 3 and 12 years after radiotherapy (mean=6.6 years). Radiotherapy was given after WLE (6 of 9), mastectomy (2 of 9) or biopsy (1 of 9). The latter patient had an inoperable tumour.

Of the nine CVD patients (*n*=9), three had no pre-existing conditions or risk factors for cardiovascular disease. Four patients had a history of hypertension, and three had received anthracycline-based chemotherapy before radiation treatment (Table 2). One of the patients had a reported history of myocardial infarction 7 years pre-radiotherapy treatment. There was no other confirmation of this patient's MI apart from a single entry in the medical records. As it may be particularly important to assess the level of deterioration in patients with pre-existing ischaemic heart disease rather than only include those who developed evidence of cardiac-related symptoms after radiotherapy, we decided to include this patient in our analysis.

All CVD patients had received left-sided radiotherapy (9 of 71) at a mean age of 65 years, and in all but one (8 of 9) the heart was within the tangential breast field (mean MHD=1.9 cm, median=0.56 and range 0–3.5 cm). The following dose-fractionation regimes were used: seven patients received 45 Gy in 20 fractions and two received 50 Gy in 25 fractions.

The comparison between the CVD patient cohort in whom the heart was within the radiation treatment field with the ‘out-of-field group’ recorded an OR of 22.3 (95% CI 2.6–185.3) (Table 3). By measuring the MHD, we had the advantage of showing that the effects of radiation are more likely to be due to direct damage to the heart rather than secondary to scattered irradiation. To test whether MHD was a significant predictor of CVD in left-sided patients, we carried out binary logistic regression, which showed that MHD shows borderline significance (*P*=0.056). There was no association between the severity of telangiectasiae and MHD in the CVD patient cohort (data not shown).

Five CVD patients who had been treated with WLE received a 15 Gy boost (Table 2). As shown previously (Giotopoulos *et al*, 2007), the use of electron boost was associated with a higher risk of telangiectasiae (Table 4). Dose per fraction (2 *vs* >2 Gy) was not a predictor for the development of CVD (data not shown). In addition, our analysis did not show an association between electron boost and the development of cardiovascular disease, as this should not give significant additional dose to the heart (Table 5). Although numbers limit the conclusions, there is some preliminary evidence that the predictive value of telangiectasiae is higher in women not receiving a boost (data not shown). The use of electron boost and hypofractionation was equal in left *vs* right patients (Table 6).

A number of factors may influence the risk and severity of late normal-tissue damage including chemotherapy or hormone therapy. Analysis of the number of patients, who received anthracycline-based adjuvant or neo-adjuvant chemotherapy regimens, showed that this was not a significant predictor for CVD in our data. There was also no association between the use of adjuvant hormone therapy (tamoxifen) and the development of CVD (data not shown).

### Right- *vs* left-sided disease and CVD

A total of 149 patients were included in this group. All of the identified patients with cardiovascular disease (9 of 9) had received radiation treatment to the left side. When the left-sided cohort was compared with the right-sided one (Table 3), a statistically significant difference in the risk for CVD was recorded (*P*=0.001; Fisher's exact test). When we carried out the exact same comparison including all documented CVD patients (without excluding patients with previous CVD), the association remained significant (11 of 71 left-sided *vs* 4 of 78 right-sided patients; *P*=0.03).

### Telangiectasiae and CVD

Presence or absence of telangiectasiae was documented for 149 patients. SOMA scores for telangiectasiae were analysed by comparing affected (SOMA scores 2–3) and unaffected patients (SOMA score 0). As in the previous study, SOMA scores of 1 (subtle, examiner-dependent changes) were excluded from analysis to try and reduce inter-examiner bias. In this cohort, 12 patients had a telangiectasiae score of 1 (denominator used in the analysis *n*=137). Thirty-two patients developed telangiectasiae SOMA score >1, of which 17 received right- and 15 received left-sided breast irradiation. Five of the thirty-two (15.6%) patients with telangiectasiae developed CVD, thus recording an OR of 6.3 (95% CI 1.4–28.0) when compared with 3 of 105 (2.9%) patients with CVD in the non-telangiectasiae group (Table 3).

When we compared excluded plus included CVD patients (without excluding any patients on the basis of pre-existing structural or functional cardiac disease) who developed telangiectasiae (SOMA score >1) (9 of 32) with the non-telangiectasiae group (6 of 105), this recorded an OR of 6.4 (95% CI 2.1–19.9) and a *P*-value of 0.004 (Fisher's exact test).

### Hypertension, smoking, diabetes and CVD

Pre-existing hypertension was reported in four of the five patients with CVD and significant telangiectasiae (SOMA >1), although this was not found to be an independent risk factor. To test this further, binary logistic regression was performed. The model contained hypertension, laterality and telangiectasia score. The model as a whole explained between 18.8 and 30% of the variance in risk for CVD and correctly classified 80.5% of cases. Telangiectasia score was the only variable that made a statistically significant contribution to the model recording an OR of 6.2 (95% CI 1.1–36.2).

Of the entire cohort, 21% of the patients were current or ex-smokers. None of the patients who developed CVD were smokers. There was also no association between a pre-existing history of diabetes and the development of CVD.

Comparison of well-established pre-existing cardiac disease risk factors in left- *vs* right-sided patients showed no differences on the basis of hypertension or age between the two groups. Smoking was slightly more common in right-sided patients (Table 7).

### Genotype–phenotype correlations

In this analysis, no association was observed between CVD and the inheritance of alleles of the *XRCC1* (R399Q) SNP, previously linked to radiation-induced telangiectasiae, the fibrosis-associated *TGFb1* (C-509T) gene promoter polymorphism (Giotopoulos *et al*, 2007) or the *eNOS* (E298D) SNP that confers a weak protective effect against radiotherapy-induced telangiectasiae (Giotopoulos *et al*, 2008).

## Discussion

Although radiotherapy has significantly increased the overall long-term survival of breast cancer patients (Clarke *et al*, 2005), normal-tissue radiation injury is increasingly becoming a factor that needs to be taken into account particularly as it can affect quality of life. In everyday clinical practice, there has also been an increasing difficulty in estimating and counselling patients about the potential adverse effects of radiation therapy and in particular their long-term risk of RIHD. In an attempt to clarify some of these issues and to assess whether there may be ways in identifying such high risk patients, we analysed data from our cohort of patients. The cardiac disease was confirmed using a variety of lines of enquiry to reduce the possibility of failing to identify patients with such diagnoses. Although we cannot say with certainty that no new cardiovascular diagnoses were missed, there should be no bias toward patients with left- and right-sided radiotherapy. Our observations indicate a statistically significant association between the long-term risk for CVD and the presence of cutaneous telangiectasiae in this cohort.

Radiation-induced heart disease is thought to result from both micro- and macrovasculature damage (Corn *et al*, 1990). The damage to the microvascular component is initiated by endothelial cell damage within cardiac structures. This is followed by ischaemia, which seems to be secondary to capillary swelling and progressive obstruction of the vessel lumen. The damaged area is then replaced by fibrous tissue. Macrovascular damage results from injury to larger vessels, leading to exacerbation of atherosclerotic lesion formation (Seddon *et al*, 2002). The most important functional consequence is diffuse interstitial myocardial fibrosis, which contributes primarily to diastolic dysfunction (Gagliardi *et al*, 2001). In our analysis, we did not observe an association between the common *TGFb1* (C-509T) gene promoter polymorphism known to significantly increase the risk for radiation-induced fibrosis and CVD. However, it is possible that other gene(s) in the same or different biological pathways may be involved in the pathogenesis of radiation-induced myocardial fibrosis. A recent study by Kuiper *et al* (2008) provides evidence for a link between vascular damage and fibrosis. It was noted that a shift in the balance between levels of the pro-angiogenic vascular endothelial growth factor and the pro-inflammatory connective tissue growth factor could explain the switch from angiogenesis to fibrosis in patients with proliferative retinopathy.

For both left- and right-sided radiotherapy and with the use of either tangential fields or intensity-modulated radiation therapy (IMRT), most of the heart receives >1 Gy dose from scattered irradiation (Li *et al*, 2000). This low-dose exposure of the whole heart may contribute to the cardiac damage leading to increase in cardiac mortality. Survivors of the atomic bombings in Hiroshima and Nagasaki also showed evidence for this. They had received a mean uniform single cardiac dose of ⩽4 Gy, and within this cohort subsequent studies showed excess mortality from cardiac disease (Preston *et al*, 2003). Another example is in men who have received treatment for germ-cell tumours. Any chemotherapy or radiotherapy to the para-aortic nodes or mediastinum in these individuals increases their cardiovascular morbidity (van den Belt-Dusebout *et al*, 2006).

It is interesting to note that in our cohort the CVD morbidity was identified after left-sided irradiation only, in spite of cardiac dose from treatment to the right side. Given the prevalence of CVD in a population with a mean age of 65 years, this finding was surprising as it is not entirely expected that only left-sided patients would have developed post-radiotherapy CVD, unless solely attributable to radiation, which seems unlikely. This may reflect a relatively small cohort.

Our data could reflect that the presence of telangiectasiae might be a better predictor for the development of cardiac disease than traditional cardiovascular risk factors, and that the mechanism precipitating this is different to that of atherosclerosis.

A number of additional factors may influence the risk and severity of late normal-tissue damage. These include, but are not limited to, the size and shape of the breast/chest wall, the type of surgery and any complications, radiotherapy dose, fractionation and time, adjuvant chemotherapy, whether the patient had a severe, acute reaction and whether the individual patient was genetically predisposed. The Standardisation of Breast radiotherapy (START) B trial has recently shown that hypofractionating regimes may not necessarily increase late effects (Bentzen *et al*, 2008). Chemotherapy may increase late effects (Fiets *et al*, 2003), particularly anthracycline-containing regimens, but we did not find a significant association between the two in our current cohort. There was also no association between the use of adjuvant hormone therapy (tamoxifen) and the development of CVD.

Telangiectasiae can develop at 6 months to many years after the completion of radiotherapy and occur most often in the boost area or the infra-mammary fold. Telangiectasiae are focal dilatations of post-capillary venules mainly, but also occasionally of the capillaries and arterioles of the subpapillary plexus (Requena and Sangueza, 1997). They occur in an atrophic dermis under a thin epidermis and present as areas of reddish discolouration (Archambeau *et al*, 1995). The mechanisms by which telangiectasiae develop are not fully understood but they seem to be both genetically and mechanistically predetermined. Their severity is influenced by both treatment- and patient-related factors. There are some fundamental radiobiological data describing macroscopic and histological effects of chronic radiation damage using animal models mainly rats (Trott, 1984). The primary effect of radiation is DNA damage and there is evidence that the most vulnerable cells are the endothelial cells. Models of telangiectasiae formation show that impaired tissue microcirculation drainage leads to stasis in the collecting venules, which results in the expansion of the capillaries and venules characteristic of telangiectasiae. In our analysis, we observed a statistically significant association between the long-term risk for cardiovascular disease and the presence of cutaneous telangiectasiae (Table 3). Interestingly, the significance of this correlation was maintained and became even greater when we included all the identified CVD patients with no exclusions. Although telangiectasiae in most cases are only unsightly, our findings could suggest a novel use as a marker and a predictor of future cardiac-related complications. The early recognition of this association may allow the clinician to modify the other cardiac risk factors in these patients and therefore decrease their risk of significant cardiac events. It could also have a function as a screening tool for earlier diagnosis and treatment in this setting.

Previous studies have shown potential common genetic and radiobiological pathways involved in the development of late normal-tissue complications after radiotherapy (Quarmby *et al*, 2003; Andreassen *et al*, 2005; Giotopoulos *et al*, 2007). A potential genotypic association between cutaneous telangiectasiae and the development of RIHD would be intriguing as it may confer a number of clinical implications. This would raise the question whether we might be able to identify patients who should be recommended to have partial breast irradiation and/or IMRT, or offer different surgical options such as a mastectomy without radiotherapy.

Our conclusions are based on a relatively small cohort and therefore these findings need to be replicated. There are a number of potential confounders that we have not been able to assess because of the design of the study, including body mass index and a family history of cardiovascular disease. A potential problem is that the cohort could have had an unexpected degree of subclinical cardiovascular disease, although the lack of previously documented CVD events in the group receiving right-sided radiotherapy mitigates against this. Although there was close consideration to accurately define the inclusion criteria for CVD diagnoses, a number of patients with additional cardiovascular events could have been lost to follow-up. The significance of the association between telangiectasiae and CVD was however maintained even with or without exclusions for previous CVD. A possible source of false association could be an undetected difference between the groups receiving left- or right-sided treatment, but in this cohort there was no difference in hypertension, telangiectasiae or other variables except CVD. Smoking was slightly more common in right-sided patients.

Our data show a statistically significant association between the development of telangiectasiae as late normal-tissue injury after radiotherapy and the risk for cardiovascular disease. The most parsimonious explanation would be that a propensity for the development of telangiectasiae and CVD are part of a common biological pathway on the basis of a genetically predisposed endothelium. If this conclusion is correct, we would expect identification of genes that prove the association. Further studies are required to explore the potential mechanisms and identification of individuals at increased risk of RIHD to maintain a reasonable therapeutic benefit for radiotherapy in breast cancer.

## Figures and Tables

**Table 1:** Summary of total radiotherapy doses including boost

**Total**	**No. of**	**No. of**	**15-Gy electron boost (No. of patients)**	
**dose (Gy)**	**fractions**	**patients**	**Boost**	**No boost**
34^a^	17	1	1	0
38^a^	17	1	1	0
40	15	9	1	8
45	20	111	65	46
50	25	27	0	27
		Total=149	Total=68	Total=81

a Patients who did not complete the planned radiotherapy schedule because of a severe early reaction to radiotherapy.

**Table 2:** Summary of patients (*n*=9) with evidence of new CVD after radiation therapy

**Patients with pre-existing conditions or risk factors for CVD (side/age at RTx)**	**Indications**	**Investigations**	**Findings (years after RTx)**	**RTx dose**
1. None (L/69)	SOB, palpitations	ECG, ECHO, CT thorax	Mild dilatation of RV and mild RV function impairment (7)	45 Gy in 20 fractions+boost
2. HT, anthracycline-based chemotherapy (L/62)	SOBOE	ECG,ECHO	Borderline LVH, CCF (5)	45 Gy in 20 fractions+boost
3. Treated SVT (L/58)	Angina, MI at 5 years post RTx	ECG, ECHO, cardiac catheterisation, coronary angiography	Mild LA dilatation, mild LAD and diagonal branch disease (5)	45 Gy in 20 fractions+boost
4. HT, anthracycline-based chemotherapy (L/66)	Palpitations	ECG, ECHO	LV dysfunction, CCF and AF (4)	50 Gy in 25 fractions
5. MI, anthracycline-based chemotherapy (L/68)	SOBOE, syncope	ECG, ECHO, myocardial perfusion scan	Mildly impaired LV function, minor fixed anterior ischaemia and possible fixed inferior ischaemia (12)	45 Gy in 20 fractions
6. HT, NIDDM (L/47)	Palpitations	ECG, ECHO	Tachy-brady syndrome, AF (11)	45 Gy in 20 fractions
7. None (L/69)	SOB	ECG, ECHO	Borderline LA dilatation (8)	45 Gy in 20 fractions+boost
8. HT (L/75)	SOBOE	ECG, ECHO	Mild LA dilatation (3)	45 Gy in 20 fractions+boost
9. None (L/78)	SOB	ECG, ECHO	Mild LVH (5)	50 Gy in 25 fractions

AF=atrial fibrillation; CCF=congestive cardiac failure; CVD=cardiovascular disease; ECG=electrocardiogram; ECHO=echocardiogram; Gy=Gray; HT=hypertension; LAD=left anterior descending artery; LA=left atrium; LVH=left ventricular hypertrophy; MI=myocardial infarction; NIDDM=non-insulin dependent diabetes mellitus; RTx=radiation therapy; RV=right ventricle; SOB=shortness of breath; SOBOE=shortness of breath on exertion; SVT=supraventricular tachycardia.

**Table 3:** Summary of results

	**Cardiac disease** a	***P*-value (test)**	**Odds ratio (95% CI)** b
*RTx Side (n*=*149)*			
Left	9/71	0.001 (Fisher's exact)	11.2 (1.3–90.6)
Right	0/78		
			
*Telangiectasiae* >*1 (n*=*137)*			
Yes (score 2–3)	5/32	0.017 (Fisher's exact)	6.3 (1.4–28.5)
No (score 0)	3/105		
			
*Field (n*=*138)*			
In	8/42	<0.001 (Fisher's exact)	22.3 (2.6–185.3)
Out	1/96		
			
*‘In-field’ and telangiectasiae* >*1 (n*=*138)*			
Yes	5/12	<0.001 (Fisher's exact)	29.3 (5.8–148.2)
No	3/126		

CI=confidence intervals; RTx=radiation therapy.

a Numbers of patients with CVD are calculated as complementary proportions of the total cohort.

b On the basis of the available information, the denominator slightly differs between the analysis groups. Treatment side information was available for 149 patients. The presence or absence of telangiectasiae was recorded for 149 patients; however, when patients with a telangiectasia score of 1 were excluded in an attempt to reduce inter-examiner bias, the analysis denominator became 137. Interpretable information through planning films could be obtained for 60 left-sided patients. The heart in right-sided patients was by definition considered as ‘out-of-treatment field’, thus giving an overall denominator for the group of 138. All the CVD patients were included in these groups.

**Table 4:** Comparison of electron boost and telangiectasiae formation in the entire cohort

	**Telangiectasia score >1 (*n*=32)**	
	**Yes**	**No**
Boost	21	41
No boost	11	64
	Total=137	
*P*-value (test)	0.008 (*χ*^2^)	

Twelve patients with a telangiectasia SOMA score of 1 (subtle, examiner-dependent changes) were excluded from analysis, thus reducing the denominator from 149 to 137. Of the excluded 12 patients, six did and six didn’t receive an electron boost.

**Table 5:** Use of electron boost and CVD

	**CVD (*n*=9)**	
	**Yes**	**No**
Boost	5	63
No boost	4	77
	Total=149	
*P*-value (test)	0.73 (Fisher's exact)	

CVD=cardiovascular disease.

**Table 6:** Use of electron boost and hypofractionation schemes in patients receiving RTx to the left *vs* the right

	**RTx side**	
**Electron Boost**	**Left**	**Right**
Yes	36	32
No	35	46
	Total=149	
*P*-value (test)	0.24 (*χ*^2^)	
		
*Dose per fraction (Gy)*		
2	14	14
>2	57	64
	Total=149	
*P*-value (test)	0.72 (*χ*^2^)	

RTx=radiation therapy; Gy=Gray.

**Table 7:** Comparison of known pre-existing cardiac risk factors in left *vs* right radiotherapy-treated patients

**RTx side**	**Mean (year) age**	**Hypertension**		**Smoking**		
**(*n*=149)**		**Yes**	**No**	**Yes**	**Ex-**	**No**
Left	55.9 (s.d. 10.8)	10	61	7	2	58
Right	57.8 (s.d. 10.0)	7	71	12	10	52
*P*-value (test)	0.36 (Mann-Whitney)	0.33 (*χ*^2^)		0.04 (*χ*^2^)		

SD=standard deviation.
